# Insulin-Like Growth Factor-1 Down-Regulates the Phosphorylation of FXYD1 and Rescues Behavioral Deficits in a Mouse Model of Rett Syndrome

**DOI:** 10.3389/fnins.2020.00020

**Published:** 2020-01-29

**Authors:** Zhe-Feng Yuan, Shan-Shan Mao, Jue Shen, Li-Hua Jiang, Lu Xu, Jia-Lu Xu, Feng Gao

**Affiliations:** Department of Neurology, The Children’s Hospital, Zhejiang University School of Medicine, National Clinical Research Center for Child Health, Hangzhou, China

**Keywords:** Rett syndrome, IGF-1, *MeCP2* mutant mice, FXYD1, neurodevelopmental disorders

## Abstract

Rett syndrome (RTT) is a neurodevelopmental disease in children that is mainly caused by mutations in the *MeCP2* gene, which codes for a transcriptional regulator. The expression of insulin-like growth factor-1 (IGF-1) is reduced in RTT patients and animal models, and IGF-1 treatment is a promising therapeutic strategy for RTT. However, the mechanism underlying the effects of IGF-1 remains to be further explored. FXYD1 is an auxiliary subunit of Na, K-ATPase. Overexpression of FXYD1 is involved in the pathogenesis of RTT. However, whether IGF-1 exerts its effect through normalizing FXYD1 is completely unknown. To this end, we evaluated the effect of IGF-1 on FXYD1 expression and posttranslational modification in a mouse model of RTT (MeCP2^308^) using both *in vitro* and *in vivo* experiments. The results show that FXYD1 mRNA and phosphorylated protein (p-FXYD1) were significantly elevated in the frontal cortex in RTT mice, compared to wild type. In RTT mice, IGF-1 treatment significantly reduced levels of FXYD1 mRNA and p-FXYD1, in parallel with improvements in behavior, motor coordination, and cognitive function. For mechanistic insight into the effect of IGF-1 on p-FXYD1, we found the decreased phosphorylated forms of PI3K-AKT-mTOR signaling pathway components in the frontal cortex of RTT mice and the normalizing effect of IGF-1 on the phosphorylated forms of these components. Interestingly, blocking the PI3K/AKT pathway by PI3K inhibitor could abolish the effect of IGF-1 on p-FXYD1 level, in addition to the effect of IGF-1 on the phosphorylation of other components in the PI3K/AKT pathway. Thus, our study has provided new insights into the mechanism of IGF-1 treatment for RTT, which appears to involve FXYD1.

## Introduction

Rett syndrome (RTT) is a serious neurodevelopmental disorder that affects young girls with an incidence of approximately 1 in 10,000 (Ip et al., 2018). RTT is characterized by normal growth and development within 6–18 months after birth, followed by regression of behaviors with progressive loss of language and hand function, autistic behaviors, stereotyped movements of the hand, and deceleration of head circumference growth. RTT is often accompanied by ataxia and convulsion, abnormal breathing, and progressive scoliosis. Severe mental retardation is common in children with RTT (Ricciardi et al., 2011; Vahdatpour et al., 2016). Previous studies have shown that RTT is caused by a decreased number of dendritic branches and dendritic spines, as well as abnormal formation, shaping, and functional transmission of synapses (Ip et al., 2018). Approximately 95% of RTT patients have X-linked *MeCP2* gene functional deletion mutations (Ip et al., 2018). *MeCP2* gene defects are also related to many serious neurodevelopmental abnormalities, such as cognitive impairment, autism, adolescent schizophrenia, and early fatal encephalopathy (Chao et al., 2010). The pathogenesis of these abnormalities is not fully understood, and there is no effective treatment. Therefore, investigating the pathogenesis and treatment of this disease is of great significance.

Insulin-like growth factor-1 (IGF-1) is an important neurotrophic factor that is widely expressed in the central nervous system (CNS) and plays an important role in the growth and development of nerve tissue. Importantly, IGF-1 can pass through the blood-brain barrier, making it possible to treat brain disorders with peripheral administration of IGF-1. IGF-1 promotes the expression of synaptic signaling pathway proteins, increases synaptic transmission, restores dendritic spine density, and effectively improves synaptic function. IGF-1 has been shown to promote the growth of neurons and glial cells through the PI3K-AKT-mTOR and MAPK-ERK pathways, which play an important role in regulating synaptic formation, maturation, and remodeling (Costales and Kolevzon, 2016; Ip et al., 2018). Previous studies have shown that there is a decrease in the PI3K-AKT-mTOR signaling pathway and endogenous IGF-1expression in a mouse model of RTT (Ricciardi et al., 2011; Castro et al., 2014). Furthermore, there is evidence that the level of IGF-1 is decreased in the cerebrospinal fluid of RTT patients (Castro et al., 2014). Supplementing with exogenous active IGF-1 peptide and recombinant human IGF-1 can improve motor function, respiration, anxiety, and other behaviors, as well as prolong the life span of RTT mice (Tropea et al., 2009; Castro et al., 2014). In clinical trials, recombinant human IGF-1 can improve abnormal respiratory movement, cognitive ability, irritability, and anxiety in RTT patients (Pini et al., 2012; Khwaja et al., 2014). Abnormal IGF-1 signaling and decreased IGF-1 levels in the cerebrospinal fluid have also been found in autism spectrum disorder (ASD) patients (Chen et al., 2014). IGF-1 treatment significantly improved motor function in a mouse model of autism (Bozdagi et al., 2013). Therefore, IGF-1 is currently considered as an ideal drug to treat a large class of neurodevelopmental disorders, including RTT and ASD (Vahdatpour et al., 2016).

FXYD domain-containing transport regulator 1 (FXYD1) is a transmembrane protein that regulates the activity of the Na, K-ATPase. The expression of FXYD1 is significantly up-regulated in the frontal cortex of RTT patients and RTT mice. Down-regulation of FXYD1 expression can reverse the neuropathological changes of RTT mice, suggesting that FXYD1 overexpression plays an important role in the pathogenesis of RTT (Deng et al., 2007; Matagne et al., 2013, 2018). However, whether IGF-1 regulates FXYD1 has not been determined. We hypothesize that the beneficial effect of IGF-1 in the treatment of RTT is mediated at least partially by normalizing FXYD1 expression or posttranslational modification (e.g., phosphorylation). Therefore, we studied the effect of IGF-1 on the neurobehavior of RTT mice using *in vitro* and *in vivo* experiments, and explored the mechanism by which IGF-1 regulates FXYD1. Our findings contribute to a better understanding of the pathophysiological mechanisms of a series of genetic neurodevelopmental disorders in children that are caused by neuronal and synaptic developmental defects, including RTT, and lay the foundation for exploring better treatment methods.

## Materials and Methods

### Animals

Experiments were carried out using the B6.129S-MeCP2tm1Hzo mouse model for RTT (MeCP2^308^ truncation model). Mecp2 hemizygous male mice and their wild-type littermates were obtained by breeding heterozygous females with wild-type C57BL/6 males. Adult heterozygous female mice were purchased from the Jackson Laboratory. Mecp2 hemizygous male mice and their wild-type littermates (5 months of age) were used instead of heterozygous female mice because of the phenotype unpredictability caused by X-chromosome inactivation in females. In cell experiments, the frontal cortex of newborn mice (postnatal day 1) was taken and cultured to obtain primary neuronal cells for subsequent experiments. All mouse procedures and experiments were approved by the Ethics Committee for Animal Experiments of Zhejiang University (ZJU20160281).

### Neuronal Preparation

For cell experiments, the newborn will-type or RTT mice on postnatal day 1 were euthanized by decapitation under anesthesia. The frontal cortices were taken and put into precooled phosphate-buffered saline (PBS) in centrifuge tubes, washed twice, and cut into small pieces. In a petri dish, the tissue pieces were further digested by adding 0.25% trypsin at room temperature and then incubated at 37°C for 5 min. Digestion was terminated by adding DMEM/F12(GIBCO, United States) with 20% fetal bovine serum (FBS). The digested tissue was further disbursed by pipetting up and down for several times. The cell suspension was centrifuged at 300 rpm for 5 min. The supernatant was discarded and replaced with neuronal culture solution, which consisted of DMEM/F12, 1% antibiotic and antimycotic, 1% glutamine, and 10% FBS. After passage through a 200-mesh filter, the cells were distributed to a 6-well plate with 1 × 10^6^ cells per well. After culturing for 6 h in the above solution, the cells were washed with DMEM/F12 at 37°C. This solution was replaced with neurobasal medium (GIBCO) supplemented with 10% FBS, B27, 1% antibiotic and antimycotic, and 1% glutamine. Cells were cultured for an additional 40 h. Then, the solution was replaced with Ara-C medium (5 μg/ml Ara-C in 1/2 neurobasal medium) to suppress the growth of glial and other cells, and further cultured to obtain the primary neuronal cells for subsequent experiments.

### Drugs and Treatment

Recombinant human IGF-1 was purchased from PeproTech Inc. (Rocky Hill, CT, United States) and freshly dissolved in normal saline prior to *in vitro* (in cultured neurons) and *in vivo* experiments. For *in vitro* experiments, six groups (*N* = 3 per group) of the cultured primary neurons from the frontal cortex were used: (1) wild-type neurons; (2) RTT neurons; (3) wild-type neurons + IGF-1 (50 ng/mL IGF-1 co-cultured for 2 days); (4) RTT neurons + IGF-1 (50 ng/mL IGF-1 co-cultured for 2 days); (5) wild-type neurons + IGF-1 + LY294002 (50 ng/mL IGF-1 and 3 × 10^–5^ mol/L LY294002 co-cultured for 2 days); (6) RTT neurons + IGF-1 + LY294002 (50 ng/mL IGF-1 and 3 × 10^–5^ mol/L LY294002 co-cultured for 2 days). The *in vivo* experiments were performed in three groups (*N* = 3 per group): (1) wild-type mice; (2) RTT mice treated with normal saline [daily intraperitoneal (i.p.) injection of normal saline for 3 weeks]; (3) RTT mice treated with IGF-1 [i.p. injection of IGF-1 (240 μg/kg/day) for 3 weeks]. Behavioral studies were conducted after the 21st i.p. injection and the subsequent 4-h washout period. After the behavioral studies, mice were sacrificed by cervical dislocation, and the frontal cortex was dissected and rapidly frozen for biochemical analyses.

### Behavioral Experiments

#### Life Behaviors

##### Hoarding of food

A single mouse was placed in a cage. The mouse was surrounded by a carton with a barbed wire attached to the side of the cage to form a 50 cm-long pipe. The mouse was provided with peanut powder at the blind end of the pipe on the outside of the cage, and water and food were supplied in the cage. After a fixed period of time, the amount of peanut powder in the cage and the amount left in the wire tube were measured.

##### Nesting

Each mouse was placed in a plastic cage with cushion materials. Six sheets of square (4 cm × 4 cm) paper were placed in the upper left corner of the cage at 20:00 h, then scored at 8:00 h the next day. The nesting score rating scale is shown in Table 1.

**TABLE 1:** Nesting scoring rating scale.

**Behavior**	**Score**
No visible cushion nests, no scraps of paper	0
Only cushion nests, no scraps of paper	1
Cushion nest, scraps of paper in or around the nest	2
Cushion nest, pieces of paper in or around the nest to form a cup-shaped fossa	3
The piece of paper covered the mouse like a ball	4

#### Motor Coordination Ability

##### Grip test

An iron wire ball was hooked with weights of 10 grades of 100 g and 190 g, respectively. The experimenter lifted the middle of the mouse tail using bare hands and measured each mouse’s claw grasp of the wire ball for 3 s, for each grade, starting from the 100-g weight level. Results were scored using: grade weight × 3 + grasp duration.

##### Grab rope

A rope was suspended in the center of the upper edge of a basin. Each mouse was lifted by the tail above the rope, then slowly lowered until it grabbed the center of the rope with its forepaws. The mouse was allowed to suspend itself beneath the rope. There was a mark along the rope every 5 cm to determine the distance that the mouse moved horizontally during suspension. The average suspension time and distance of three experiments were recorded. The conversion points were calculated as: average suspension time + 10 × average distance.

##### Balance beam

A 160-cm iron rod with diameter of 0.6 cm was fixed on the white iron basin. Mouse movement was observed for 60 s. The duration for which each mouse maintained balance was measured three times. Table 2 shows the balance beam score rating scale.

**TABLE 2:** Balance beam scoring rating scale.

**Behavior**	**Score**
Don’t fall through the balance beam	0
Less than 50% chance of falling	1
A greater than 50% chance of falling	2
Can cross the balance beam, but slowly	3
Can’t go through the balance beam, but can stay on the pole	4
Fall off the balance beam	5

#### Cognitive Function

##### Hidden platform

A Morris water maze was constructed at the Chinese Academy of Medical Sciences. The pool wall was marked with four points for water entry in the southeast and northwest corners. The pool was equally divided into four quadrants. A circular hidden platform with diameter of 11 cm and height of 29 cm was placed in the center of the target quadrant. Its position remained unchanged throughout the experiment. Milk (1000 mL) was added to the warm (25°C) water in the pool to hide the platform. The references outside the maze remained unchanged throughout the experiment. A camera with a display was placed above the maze and connected to a video recorder to automatically track swimming. Morris water maze data acquisition and analysis software was used to record the relevant data and image results. The mice were placed in the water facing the pool wall, and the time it took the mouse to find and mount the platform was recorded as escape latency. If the mouse did not find the platform within 120 s, the experimenters rescued the mice to the platform. In this case, escape latency was recorded as 120 s.

##### Spatial search

On the fifth day of the water maze experiments, the platform was removed, and the mice were put into the water at any point of entry. The swimming path was recorded for 120 s. The observed results were statistically processed. The swimming path score rating scale is shown in Table 3.

**TABLE 3:** Swimming path scoring rating scale.

**Path**	**Score**
Linear form	4
Curve form	3
Random form	2
Circle form	1

### Western Blot

Western blotting was performed as previously described (Yuan et al., 2016). Briefly, the obtained cells and frontal cortex tissues were lysed with lysis buffer [50 mM Tris–HCl (pH 7.5), 150 mM NaCl, 1% NP-40, 0.5% sodium deoxycholate, 0.1% sodium dodecyl sulfate (SDS), 1 mM EDTA, 1 mM phenylmethylsulfonyl fluoride, and protease inhibitors] for 10 min on ice. After centrifugation at 12,000 × *g* for 10 min at 4°C, the supernatant was transferred to a fresh tube and stored at -70°C. Protein concentrations were determined using a bicinchoninic acid protein assay (Beyotime Institute of Biotechnology, Shanghai, China). Samples containing 50 μg of protein were electrophoresed in a 10% SDS-polyacrylamide gel and subsequently transferred onto polyvinylidene fluoride membranes (Merck Chemicals Co., Ltd., Shanghai, China). The membranes were blocked with 5% non-fat milk in Tris-buffered saline for 2 h at room temperature, followed by incubation with primary antibodies against AKT (1:500; Proteintech Group, Inc., Chicago, IL, United States), p-AKT (Ser473) (1:500, Affinity Biosciences, OH, United States), p-mTOR (Ser2448) (1:500; Affinity Biosciences, OH, United States), p-S6 (Ser235/236) (1:2000, Santa Cruz Biotechnology, Inc., Dallas, TX, United States), FXYD1 (1:500, Proteintech Group, Inc., Chicago, IL, United States), p-FXYD1 (Ser83) (1:500, Bioss, Beijing, China) and GAPDH (1:1000, Santa Cruz Biotechnology, Inc., Dallas, TX, United States) in Tris-buffered saline at 4°C overnight. After washing, the membranes were incubated with secondary antibody [horseradish peroxidase (HRP)-conjugated goat anti-mouse or anti-rabbit immunoglobulin IgG antibody; 1:2000; Santa Cruz Biotechnology, Inc., Dallas, TX, United States] for 2 h at room temperature. The blotting bands were visualized using an enhanced chemiluminescence (ECL) system (ECL kit; Beyotime Institute of Biotechnology), quantified by densitometry, and normalized to GAPDH using Image Pro Plus software (Media Cybernetics, Inc., Rockville, MD, United States).

### Real-Time Polymerase Chain Reaction (PCR)

For total RNA extraction, the frontal cortex tissues were homogenized in TriZol reagent (Invitrogen, Carlsbad, CA, United States), purified with chloroform and precipitated with isopropanol, then washed with ethanol, and dissolved in RNAse-free water. For reverse transcription, cDNA was synthesized using a First-Strand cDNA Synthesis Kit (Thermo Fisher, Waltham, MA, United States) according to the manufacturer’s instructions. SYBR Green Realtime PCR Master Mix (TOYOBO Co., Ltd., Osaka, Osaka Prefecture, Japan) was used to perform quantitative real-time PCR. All real-time PCR reactions were performed using samples collected from at least three independent preparations. All data were normalized by standardizing to an endogenous control: GADPH. The primer sequences used for this study were as follows: 5′-GTGCACAGCTGGACATTTGG-3′ (forward) and 5′-ACACACACAGAGCCAGGATG-3′ (reverse) for FXYD1; 5′-TATGTCGTGGAGTCTACTGGT-3′ (forward) and 5′-GAGTTGTCATATTTCTCGTGG-3′ (reverse) for GADPH.

### Statistical Analysis

Behavioral experiments were analyzed using the non-parametric test, Kruskal–Wallis and Chi square methods, with Bonferroni test for pairwise comparison. Data were analyzed using SAS software (version 9.0; SAS Institute Inc., United States). One-way analysis of variance (one-way ANOVA) was applied to determine the difference in biochemical markers among different groups of treatments and controls. The Fisher’s Least Significant Difference (LSD) test was used as *post hoc* test for one-way ANOVA. Data were analyzed using SPSS software (version 18.0; SPSS Inc., Chicago, IL, United States). Data are presented as the mean ± standard deviation. *P* < 0.05 was considered to indicate a statistically significant difference.

## Results

### IGF-1 Treatment Improves the Behavior, Motor Coordination, and Cognitive Function of RTT308 Mice

Although it has been reported that IGF-1 treatment can improve these parameters in patients and in some animal models, because there are at least 22 animal models with different mutations or deletions of the MeCP2 gene, and differences in age, gender, and female X chromosome inactivation, the phenotype and effect of IGF-1 could vary significantly. Thus, it is necessary to first observe the difference in behavior of the wild-type and mutant mice, and the effect of IGF-1 in our animal model.

Table 4 shows the results of food hoarding and nesting ability of animals in each group. The results showed that food hoarding of RTT mice were significantly decreased compared to the wild-type group (*p* < 0.05), but the nesting ability had no significant difference. After IGF-1 treatment, food hoarding of the RTT mice was significantly enhanced (*p* < 0.05).

**TABLE 4:** The results of life behavior test.

**Group**	**Food hoarding(g)**			**Nesting**		
	**(1)**	**(2)**	**(3)**	**(1)**	**(2)**	**(3)**
Wild type	11.4	15	13	3	3	3
RTT + NS	2.0	1.5	1.7	2	1	2
RTT + IGF-1	10.4	10.9	10	3	2	3

*Food hoarding: Group (Wild type) vs. Group (RTT + NS), *p* < 0.05; Group (RTT + NS) vs. Group (RTT + IGF-1), *p* < 0.05. Nesting: Group (Wild type) vs. Group (RTT + NS), *p* > 0.05; Group (RTT + NS) vs. Group (RTT + IGF-1), *p* > 0.05. NS, normal saline.*

Table 5 shows the results of motor coordination ability of animals in each group. Compared with the wild-type mice, the grasping strength and rope grasping ability of RTT mice were significantly decreased (both *p* < 0.05), and the balance beam test had no significant difference. After IGF-1 treatment, the grasping strength and rope grasping ability increased significantly in the RTT mice (both *p* < 0.05), indicating that IGF-1 significantly enhanced motor coordination.

**TABLE 5:** The results of motor coordination ability test.

**Group**	**Grisp strength**			**Grab rope**			**Balance beem**		
	**(1)**	**(2)**	**(3)**	**(1)**	**(2)**	**(3)**	**(1)**	**(2)**	**(3)**
Wild type	24	23	25	96	109	116	0.3	0.3	0.3
RTT + NS	10	8	9	27	43	41	4	3	3
RTT + IGF-1	19	15	20	56	60	73	1.7	0	0.7

*Grisp strength: Group (Wild type) vs. Group (RTT + NS), *p* < 0.05; Group (RTT + NS) vs. Group (RTT + IGF-1), *p* < 0.05. Grab rope: Group (Wild type) vs. Group (RTT + NS), *p* < 0.05; Group (RTT + NS) vs. Group (RTT + IGF-1), *p* < 0.05. Balance beem: Group (Wild type) vs. Group (RTT + NS), *p* > 0.05; Group (RTT + NS) vs. Group (RTT + IGF-1), *p* > 0.05. NS, normal saline.*

Table 6 shows the results of cognitive function of animals in each group. Compared with the wild-type mice, the time to find the hidden platform was significantly increased (*p* < 0.05) in the RTT mice, and the spatial search ability had no significant difference. IGF-1 treatment significantly reversed time to find the platform (*p* < 0.05) in the RTT mice.

**TABLE 6:** The results of cognitive function test.

**Group**	**Platform access(s)**			**Spatial search**		
	**(1)**	**(2)**	**(3)**	**(1)**	**(2)**	**(3)**
Wild type	26	18	24	3	3	3
RTT + NS	51	63	49	2	2	1
RTT + IGF-1	36	45	38	3	2	2

*Platform access: Group (Wild type) vs. Group (RTT + NS), *p* < 0.05; Group (RTT + NS) vs. Group (RTT + IGF-1), *p* < 0.05. Spatial search: Group (Wild type) vs. Group (RTT + NS), *p* > 0.05; Group (RTT + NS) vs. Group (RTT + IGF-1), *p* > 0.05. NS, normal saline.*

The above studies have confirmed that living behavior, motor coordination, and cognitive function were significantly decreased in our RTT mice model and that the IGF-1 treatment improved these behaviors. Our findings indicate that IGF-1 has good therapeutic potential for improving behavior in this RTT mice model.

### IGF-1 Treatment Decreases mRNA Expression of FXYD1 but Does Not Change Its Protein Expression in the Frontal Cortex

**FIGURE 1:**
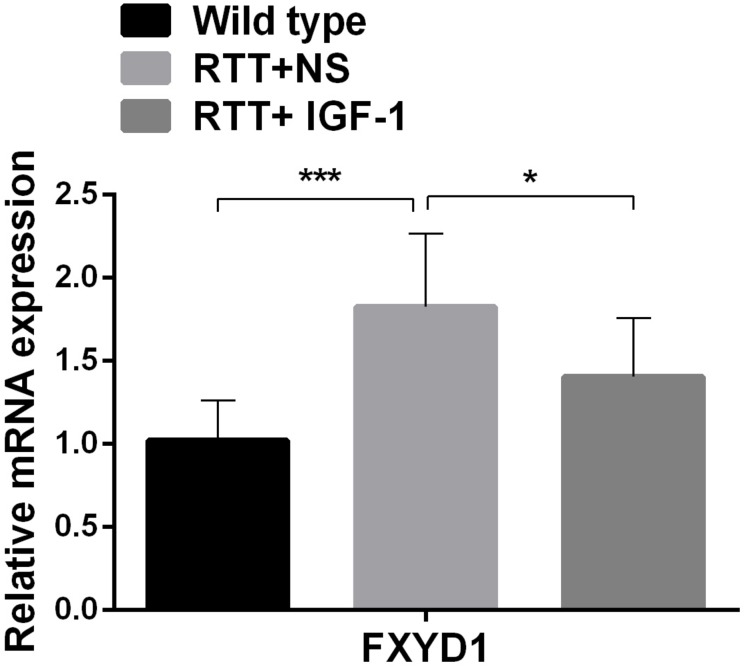
IGF-1 treatment decreases mRNA expression of FXYD1. FXYD1 mRNA expression level in the frontal cortex was measured by Real-Time PCR. Compared to the wild-type mice, FXYD1 mRNA level in the frontal cortex of the RTT mice was increased significantly (*p* < 0.001). However, after treatment with IGF-1 for 3 weeks, the mRNA level of FXYD1 in the RTT mice was significantly decreased (*p* < 0.05). Data was presented as mean values ± SD (*N* = 3) (*p < 0.05, ***p < 0.001).

To further explore the mechanism of IGF-1 effect and to test our hypothesis that the beneficial effect of IGF-1 treatment on RTT is mediated at least partially through normalizing FXYD1 expression or functional state, we observed the effect of IGF-1 on the expression of FXYD1 in the frontal cortex. First, we quantitated the mRNA expression level for FXYD1 by reverse transcription and real-time quantitative PCR. Figure 1 shows the FXYD1 mRNA levels in the frontal cortex of each group of mice. Compared with the wild-type mice, FXYD1 mRNA levels increased significantly in the RTT mice (*p* < 0.001). This is consistent with the previous finding (Deng et al., 2007; Matagne et al., 2013). Notably, IGF-1 treatment significantly decreased the FXYD1 mRNA level in the RTT mice (*p* < 0.05).

**FIGURE 2:**
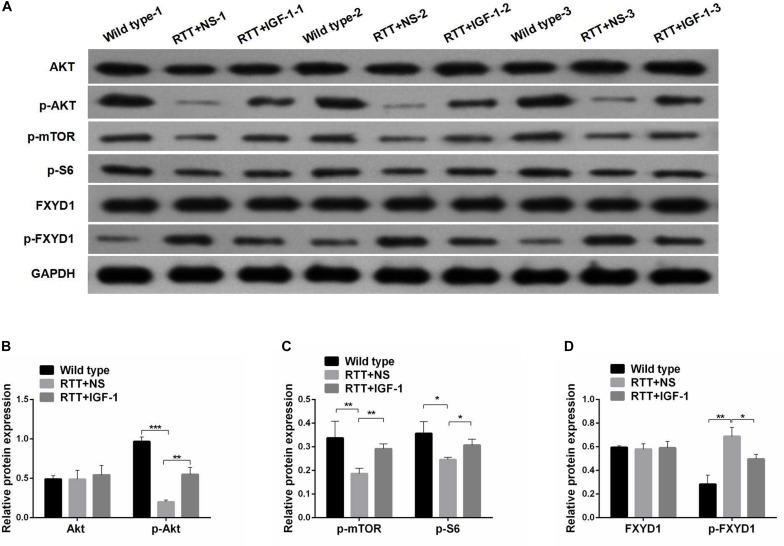
Protein phosphorylation for FXYD1 and PI3K/AKT pathway components in the frontal cortex of RTT mice is altered, and IGF-1 treatment reverses the change. **(A)** Western blot analysis of the expression of FXYD1 and p-FXYD1 along with AKT, p-AKT, p-mTOR, and p-S6 in the frontal cortex of the wild-type mice and the RTT mice treated with normal saline (NS) or IGF-1 for 3 weeks. **(B)** Quantitative analysis of the blots shown in **(A)** for AKT and p-AKT in all three groups. **(C)** Quantitative analysis of the blots shown in **(A)** for p-mTOR, and p-S6 in all three groups. **(D)** Quantitative analysis of the blots shown in **(A)** for FXYD1 and p-FXYD1 in all three groups (*p < 0.05, **p < 0.01, ***p < 0.001). Note that the p-FXYD1 in the RTT mice was significantly higher than that in the wild-type control, and IGF-1 treatment normalized the p-FXYD level in the RTT mice. In contrast, the levels of p-AKT, p-mTOR, and p-S6 were significantly decreased in the RTT mice compared to the wild-type, and IGF-1 treatment significantly reversed the under-phosphorylation of these proteins.

**FIGURE 3:**
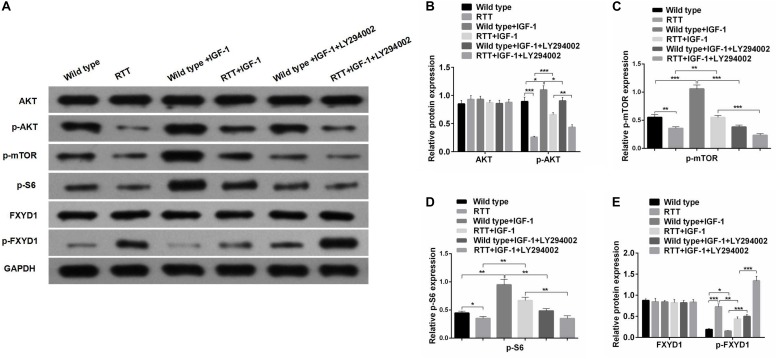
IGF-1 effect on FXYD1 phosphorylation is abolished by blocking PI3K/AKT signaling pathway. **(A)**Western blot analysis of the protein levels of AKT, p-AKT, p-mTOR, p-S6, FXYD1, and p-FXYD1 in the cultured primary neurons from the frontal cortex of the RTT mice or the wild-type control without or with the treatment of IGF-1 (50 ng/mL IGF-1 co-cultured for 2 days) or IGF-1 (50 ng/mL) + LY294002 (3 × 10^– 5^ M) co-cultured for 2 days. **(B)** Quantitative analysis of the blot results from **(A)** for expression of AKT and p-AKT. **(C)** Quantitative analysis of the blot results from **(A)** for expression of p-mTOR. **(D)** Quantitative analysis of the blot results from **(A)** for expression of p-S6. **(E)** Quantitative analysis of the blot results from **(A)** for expression of FXYD1 and p-FXYD1 (*p < 0.05, **p < 0.01, ***p < 0.001). Note that similar to the *in vivo* experiment as shown in Figure 2, the p-FXYD1 level in the cultured primary neurons from frontal cortex of the RTT mice was also elevated when compared to the wild type. Again, IGF-1 treatment significantly reduced the p-FXYD1 level. However, the effect of IGF-1 on p-FXYD1 was abolished by the PI3K inhibitor LY294002. In addition, the decreases in the expression of p-AKT, p-mTOR, and p-S6 in the RTT neurons, compared to the wild-type control, were also consistent with *in vivo* results. Notably, LY94002 treatment resulted in reduction of these phosphorylated forms of the PI3K-AKT-mTOR signaling pathway components.

Next, we evaluated the effect of IGF-1 on the expression of FXYD1 at protein level. Figures 2A,D shows that the total protein level of FXYD1 in the frontal cortex of the RTT mice was not significantly different from that of the wild-type mice. Furthermore, The IGF-1 treatment did not significantly change the FXYD1 protein expression level in the frontal cortex of the RTT mice. Similarly, in the primary culture neurons of RTT mice, the FXYD1 protein expression was not significantly different from that in the wild-type neurons (Figures 3A,E).

### Phosphorylated FXYD1 Protein in the Frontal Cortex of RTT Mice Is Increased and IGF-1 Treatment Reduces the Phosphorylated Form of FXYD1

FXYD1 protein is also subjected to posttranslational modification. Specifically, four serine or threonine residues of its C-terminal intracellular domain can be phosphorylated by protein kinase A or protein kinase C (Walaas et al., 1994). Interestingly, phosphorylation of these residues can neutralize ER retention signals and help FXYD1’s membrane targeting (Lansbery et al., 2006). Thus, even if the total protein of FXYD1 in RTT mice was not different from the wild-type and was not changed by IGF-1 treatment in RTT mice, it is still possible that its membrane fraction, the final functional form, can be different and can be modulated through IGF-1 signaling. To test this hypothesis, we used a phosphorylation specific antibody to detect the phosphorylated form of FXYD1 (p-FXYD1 at Ser82) in the frontal cortex in the RTT mice and compared it to the wild-type control. Figures 2A,D shows that p-FXYD1 was significantly elevated in the RTT mice compared to the wild-type control (*p* < 0.01), and IGF-1 treatment reduced the p-FXYD1 level (*p* < 0.05). Similarly, in the primary cultured neurons, p-FXYD1 was about threefold higher in the RTT mice-derived neurons than that from the wild-type control. Again, IGF-1 treatment significantly reduced p-FXYD1 level in the RTT neurons (Figures 3A,E). Thus, IGF-1 treatment can reduce membrane targeting of FXYD1.

### IGF-1 Effect on FXYD1 Phosphorylation Is Abolished by Blocking PI3K/AKT Signaling Pathway

To further investigate the mechanism underlying the effect of IGF-1 on FXYD1 phosphorylation, we examined IGF-related PI3K-AKT-mTOR signaling pathway in the cultured primary neurons from the frontal cortex and RTT mice. The total protein level of AKT in RTT mice was similar to that in the control, and IGF-1 treatment did not have any significant effect on AKT level (Figures 2A,B). However, phosphorylated AKT (p-AKT) was dramatically decreased in the RTT mice compared to the control. IGF-1 treatment significantly elevated p-AKT level in the RTT mice (Figures 2A,B). Similar changes in p-AKT were observed in the cultured primary neurons (Figures 3A,B). We then further examined the phosphorylated forms of other components in this signaling pathway. Figures 2A,C, 3A,C,D show that p-mTOR, and p-S6 protein expression in both frontal cortex and cultured primary neurons was significantly reduced in RTT model compared to control, and IGF-1 treatment significantly normalized their levels. Interestingly, the PI3K inhibitor, LY294002, abolished the effect of IGF-1 on the phosphorylated forms of these proteins (Figure 3A), including p-FXYD1. Thus, IGF-1 effect on the phosphorylation of FXYD1 is dependent on the PI3K/AKT dependent signaling pathway.

## Discussion

*MeCP2* is the main pathogenic gene of RTT. The encoded MeCP2 protein inhibits gene transcription, RNA splicing, miRNA expression, and transcriptional activation (Ip et al., 2018). *MeCP2* gene mutations can directly lead to abnormal methylation of downstream target genes, interfere with normal brain development, and eventually lead to abnormal synaptic formation, maturation, and shaping of the nervous system. *MeCP2* mutations can also cause dysfunctional neural networks (Ip et al., 2018). The expression of IGF-1 has been found to be decreased in the CNS of RTT, and administration of IGF-1 is a promising treatment for RTT. However, the mechanisms of IGF-1 treatment in RTT is still not completely clear. On the other hand, the expression of FXYD1 in the frontal cortex of RTT patients and RTT mice was significantly upregulated. Since FXYD1 can inhibit the activity of Na, K-ATPase, overexpression of FXYD1 can cause typical neuropathological manifestations of RTT, such as a reduction in dendritic branches and dendritic spines. In contrast, down-regulation or knockout of FXYD1 can reverse neuropathological changes in RTT mice. These studies suggest that overexpression of FXYD1 plays an important role in the pathogenesis of RTT and could serve as important therapeutic target (Deng et al., 2007; Matagne et al., 2013, 2018). However, it is completely unknown that whether IGF-1 exerts its effect on RTT through regulating FXYD1.

To this end, we have provided the experimental evidence to support our hypothesis that IGF-1’s beneficial effect on RTT is at least partially through inhibiting expression of FXYD1. In this study, we first confirmed the beneficial effect of IGF-1 on neurobehavior parameters in our experimental condition. We found that IGF-1 significantly improved living behaviors, motor coordination, and cognitive function of RTT mice, suggesting that IGF-1 has therapeutics effects for improving neurobehaviors in our RTT mice model. This conclusion is consistent with previous studies (Tropea et al., 2009; Castro et al., 2014). We then demonstrated that IGF-1 treatment can inhibit mRNA expression and protein phosphorylation of FXYD1. Since phosphorylation of FXYD1 is important for its membrane targeting, our result suggests that IGF-1 can influence FXYD1 membrane targeting. Furthermore, we have demonstrated that IGF-1’s effect on the phosphorylation of FXYD1 is mediated through PI3K-AKT-mTOR signaling pathway. IGF-1 treatment normalized the phosphorylation of the important components of this pathway. Finally, the pathway inhibitor at PI3K level can abolish the effect of IGF-1 on the phosphorylation of FXYD1 and other pathway components. Thus, we have found, for the first time, that IGF-1 treatment has an important impact on the posttranslational modification of FXYD1, and we have provided further evidence for a mechanistic link between IGF-1 receptor activation and the regulation of FXYD1 phosphorylation.

IGF-1 plays an important role in the development and maturation of the CNS through the PI3K-AKT-mTOR and MAPK-ERK signaling pathways (Costales and Kolevzon, 2016; Ip et al., 2018). However, the roles of other pathways remain to be explored. Previous studies have suggested that there was abnormal PI3K-AKT-mTOR signaling in developmental disorders such as RTT and ASD (Chen et al., 2014), and the level of endogenous IGF-1 was decreased in RTT mice. Similarly, IGF-1 levels were decreased in the cerebrospinal fluid of RTT patients (Castro et al., 2014). The phosphorylation level of PI3K-AKT-mTOR signaling pathway was decreased in RTT mice, and the phosphorylation level of rpS6 (the ribosomal protein S6) which was a downstream molecule of mTOR, was also decreased in RTT mice (Ricciardi et al., 2011). Decreased PI3K-AKT-mTOR signaling is one of the pathological features of RTT. Our study confirmed that p-AKT, p-mTOR, and p-S6 expression in RTT neurons was significantly lower compared to the wild-type neurons *in vitro*. IGF-1 significantly increased p-AKT, p-mTOR, and p-S6 in RTT neurons compared to before treatment. The effect of IGF-1 was reversed by the PI3K inhibitor LY294002. Our *in vivo* analysis showed that p-Akt, p-mTOR, and p-S6 expression in the frontal cortex of RTT mice were significantly lower compared to the wild-type mice. IGF-1 significantly increased p-Akt, p-mTOR, and p-S6 expression in the brain tissue of RTT mice. Our study further confirmed that IGF-1 can improve neuronal and synaptic function and clinical symptoms of RTT through modulating the PI3K-AKT-mTOR-S6 signaling pathway, consistent with previous studies (Costales and Kolevzon, 2016; Ip et al., 2018).

**FIGURE 4:**
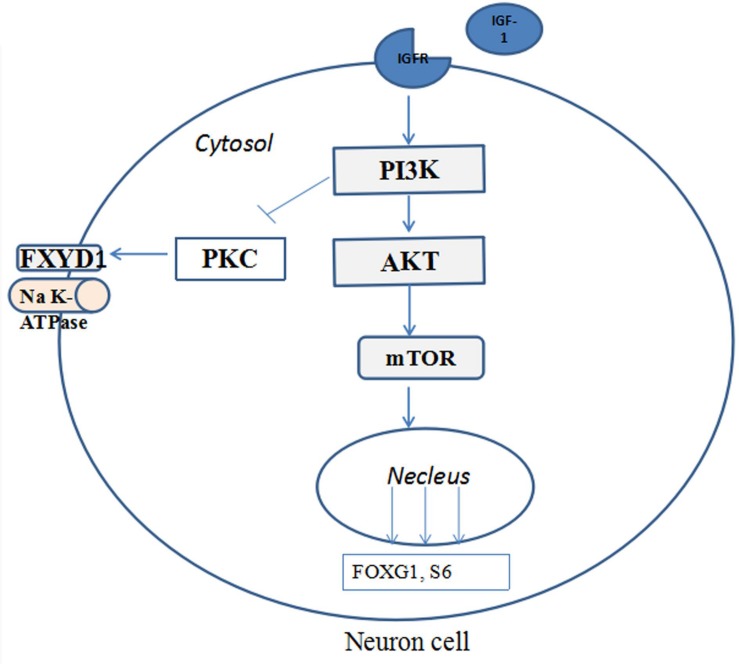
The potential mechanisms of IGF-1 treatment on the phosphorylation of FXYD1 through PI3K/AKT pathway. Binding of IGF-1 to its receptor activates the PI3K-AKT-mTOR pathway and phosphorylates the pathway components and inhibition of FXYD1 phosphorylation.

Figure 4 illustrates the potential mechanism of IGF-1 treatment on the phosphorylation of FXYD1 through PI3K-AKT-mTOR pathway. Specifically, activation of PI3K by IGF-1 receptor can lead to suppression of PKC. Although detailed molecular mechanism still needs to be elucidated, two pathway components are likely involved in mediating the PKC inhibition through PI3K activation. First, previous studies have shown that PKC plays a negative feedback role in IGF-1-induced AKT activation and can inhibit AKT phosphorylation (Zheng et al., 2000; Shahaf et al., 2012; Dowling et al., 2016). There may be mutual inhibition between PKC and AKT. In our experiment, IGF-1 significantly increased p-Akt in RTT neurons and mice. The increased p-Akt may inhibit PKC, for which FXYD1 is a substrate, so phosphorylation of FXYD1 decreases with PKC inhibition. The PI3K inhibitor, LY294002, significantly increased p-FXYD1 and inhibited AKT phosphorylation in RTT neurons treated with IGF-1, which further confirms that IGF-1 may reduce p-FXYD1 through the PI3K/AKT-PKC pathway. Thus, in the wild-type animal, the expression of IGF-1 is normal, and activation of the PI3K-AKT-mTOR pathway would cause inhibition of PKC. In the RTT condition, however, insufficient IGF-1 level will cause poor activation of this pathway and under phosphorylation of the AKT, which in turn reduces the inhibitory effect on PKC, resulting in over phosphorylation of FXYD1. In addition, Tropea et al. (2016) found that IGF-1 could increase *MeCP2* mRNA levels and the nuclear localization of MeCP2 protein, increase the copy number of wild-type *MeCP2*, and improve the symptoms of RTT. Therefore, IGF-1 may increase the wild-type *MeCP2* level, at least in some chimeric condition, resulting in the inhibition of the FXYD1 gene transcription. The exact molecular mechanism for the beneficial effect of IGF-1 on RTT needs to be further explored, for example using selective inhibitors of the pathway components downstream of PI3K.

FXYD1 is a transmembrane protein that regulates Na, K-ATPase activity and is the target gene of transcriptional inhibition of *MeCP2* (Deng et al., 2007). FXYD1 can be phosphorylated by protein kinase A (PKA), protein kinase C (PKC), myotonic dystrophy protein kinase (DMPK), and other kinases, and plays an important role in maintaining the excitability of neurons (Matagne et al., 2018). The detailed mechanism of our finding that IGF-1 treatment can reduce p-FXYD1 still need to be explored. Although we speculate the effect of IGF-1 on FXYD1 phosphorylation is through inhibiting PKC, we cannot exclude other possibilities through different protein kinases, such as PKA. In fact, Ser82 in FXYD1 (the antibody used in our study to detect the phosphorylated form of FXYD1 is for this site) is the substrate of both PKA and PKC. It is also interesting to explore the direct impact of the phosphorylation of FXYD1 on different subtypes of Na, K-ATPase with different isoforms of α and β subunits and final outcome to the neuronal development and detailed molecular mechanism.

It should be noted that the major limitation was only three animals used for each group in this study, and this precluded us to examine the animal behaviors comprehensively. Future studies with large number sample size of animals are necessary to systemically evaluate the role of IGF-1/FXYD1 axis in regulating behavior deficits of RTT.

In conclusion, we found, for the first time, that IGF-1 can relieve the symptoms of RTT by down-regulating the phosphorylation level of FXYD1, which provides a new therapeutic mechanism for a large class of neurodevelopmental disorders. The molecular mechanism underlying these therapeutic effects warrants further study, and it might be helpful to explore mechanism-based treatments in the future.

## Data Availability Statement

All datasets generated for this study are included in the article/supplementary material.

## Ethics Statement

All mice procedures and experiments were approved by the Ethics Committee for Animal Experiments of Zhejiang University (with approved protocol number of ZJU20160281) and were performed in accordance with the guidelines of the National Institutes of Health on the Care and Use of Animals.

## Author Contributions

FG contributed to the study design. Z-FY drafted the manuscript. S-SM, JS, L-HJ, LX, and J-LX helped to draft the manuscript. All authors read and approved the final manuscript.

## Conflict of Interest

The authors declare that the research was conducted in the absence of any commercial or financial relationships that could be construed as a potential conflict of interest.

## References

[B1] Bozdagi O. Tavassoli T. Buxbaum J. D. (2013). Insulin-like growth factor-1 rescues synaptic and motor deficits in a mouse model of autism and developmental delay. *Mol. Autism.* 4:9. 10.1186/2040-2392-4-9 23621888 PMC3649942

[B2] Castro J. Garcia R. I. Kwok S. Banerjee A. Petravicz J. Woodson J. (2014). Functional recovery with recombinant human IGF1 treatment in a mouse model of Rett syndrome. *Proc. Natl. Acad. Sci. U.S.A.* 111 9941–9946. 10.1073/pnas.1311685111 24958891 PMC4103342

[B3] Chao H. T. Chen H. Samaco R. C. Xue M. Chahrour M. Yoo J. (2010). Dysfunction in GABA signalling mediates autism-likestereotypies and Rett syndrome phenotypes. *Nature* 468 263–269. 10.1038/nature09582 21068835 PMC3057962

[B4] Chen J. Alberts I. Li X. (2014). Dysregulation of the IGF-I/PI3K/AKT/mTOR signaling pathway in autism spectrum disorders. *Int. J. Dev. Neurosci.* 35 35–41. 10.1016/j.ijdevneu.2014.03.006 24662006

[B5] Costales J. Kolevzon A. (2016). The therapeutic potential of insulin-like growth factor-1 in central nervous system disorders. *Neurosci. Biobehav. Rev.* 63 207–222. 10.1016/j.neubiorev.2016.01.001 26780584 PMC4790729

[B6] Deng V. Matagne V. Banine F. Frerking M. Ohliger P. Budden S. (2007). FXYD1 is an MeCP2 target gene overexpressed in the brains of Rett syndrome patients and Mecp2-null mice. *Hum. Mol. Genet.* 16 640–650. 10.1093/hmg/ddm007 17309881

[B7] Dowling C. M. Phelan J. Callender J. A. Cathcart M. C. Mehigan B. McCormick P. (2016). Protein kinase C beta II suppresses colorectal cancer by regulating IGF-1 mediated cell survival. *Oncotarget* 7 20919–20933. 10.18632/oncotarget.8062 26989024 PMC4991501

[B8] Ip J. P. K. Mellios N. Sur M. (2018). Rett syndrome: insights into genetic, molecular and circuit mechanisms. *Nat. Rev. Neurosci.* 19 368–382. 10.1038/s41583-018-0006-3 29740174 PMC6402579

[B9] Khwaja O. S. Ho E. Barnes K. V. O’Leary H. M. Pereira L. M. Finkelstein Y. (2014). Safety, pharmacokinetics, and preliminary assessment of efficacy of mecasermin (recombinant human IGF-1) for the treatment of Rett syndrome. *Proc. Natl. Acad. Sci. U.S.A.* 111 4596–4601. 10.1073/pnas.1311141111 24623853 PMC3970488

[B10] Lansbery K. L. Burcea L. C. Mendenhall M. L. Mercer R. W. (2006). Cytoplasmic targeting signals mediate delivery of phospholemman to the plasma membrane. *Am. J. Physiol. Cell Physiol.* 290 C1275–C1286. 16371442 10.1152/ajpcell.00110.2005

[B11] Matagne V. Budden S. Ojeda S. R. Raber J. (2013). Correcting deregulated Fxyd1 expression ameliorates a behavioral impairment in a mouse model of Rett syndrome. *Brain Res.* 1496 104–114. 10.1016/j.brainres.2012.12.009 23246925 PMC3556227

[B12] Matagne V. Wondolowski J. Frerking M. Shahidullah M. Delamere N. A. Sandau U. S. (2018). Correcting deregulated Fxyd1 expression rescues deficits in neuronal arborization and potassium homeostasis in MeCP2 deficient male mice. *Brain Res.* 1697 45–52. 10.1016/j.brainres.2018.06.013 29902467 PMC7864099

[B13] Pini G. Scusa M. F. Congiu L. Benincasa A. Morescalchi P. Bottiglioni I. (2012). IGF1 as a potential treatment for Rett syndrome: safety assessment in six Rett patients. *Autism. Res. Treat.* 2012:679801. 10.1155/2012/679801 22934177 PMC3420537

[B14] Ricciardi S. Boggio E. M. Grosso S. Lonetti G. Forlani G. Stefanelli G. (2011). Reduced AKT/mTOR signaling and protein synthesis dysregulation in a Rett syndrome animal model. *Hum. Mol. Genet.* 20 1182–1196. 10.1093/hmg/ddq563 21212100

[B15] Shahaf G. Rotem-Dai N. Koifman G. Raveh-Amit H. Frost S. A. Livneh E. (2012). PKCη is a negative regulator of AKT inhibiting the IGF-I induced proliferation. *Exp. Cell Res.* 318 789–799. 10.1016/j.yexcr.2012.01.018 22305966

[B16] Tropea D. Giacometti E. Wilson N. R. Beard C. McCurry C. Fu D. D. (2009). Partial reversal of Rett syndrome-like symptoms in MeCP2 mutant mice. *Proc. Natl. Acad. Sci. U.S.A.* 106 2029–2034. 10.1073/pnas.0812394106 19208815 PMC2644158

[B17] Tropea D. Mortimer N. Bellini S. Molinos I. Sanfeliu A. Shovlin S. (2016). Expression of nuclear Methyl-CpG binding protein 2 (Mecp2) is dependent on neuronal stimulation and application of Insulin-like growth factor 1. *Neurosci. Lett.* 621 111–116. 10.1016/j.neulet.2016.04.024 27080430

[B18] Vahdatpour C. Dyer A. H. Tropea D. (2016). Insulin-Like Growth Factor 1 and related compounds in the treatment of childhood-onset neurodevelopmental disorders. *Front. Neurosci.* 10:450. 10.3389/fnins.2016.00450 27746717 PMC5043261

[B19] Walaas S. I. Czernik A. J. Olstad O. K. Sletten K. Walaas O. (1994). Protein kinase C and cyclic AMP-dependent protein kinase phosphorylate phospholemman, an insulin and adrenaline-regulated membrane phosphoprotein, at specific sites in the carboxy terminal domain. *Biochem. J.* 304 635–640. 10.1042/bj3040635 7999001 PMC1137538

[B20] Yuan Z. F. Tang Y. M. Xu X. J. Li S. S. Zhang J. Y. (2016). 10-Hydroxycamptothecin induces apoptosis in human neuroblastoma SMS-KCNR cells through p53, cytochrome c and caspase 3 pathways. *Neoplasma* 63 72–79. 10.4149/neo_2016_009 26639236

[B21] Zheng W. H. Kar S. Quirion R. (2000). Stimulation of Protein Kinase C Modulates Insulin-like Growth Factor-1-induced Akt Activation in PC12 Cells. *J. Biol. Chem.* 275 13377–13385. 10.1074/jbc.275.18.13377 10788447

